# Computational Chemical Imaging for Cardiovascular Pathology: Chemical Microscopic Imaging Accurately Determines Cardiac Transplant Rejection

**DOI:** 10.1371/journal.pone.0125183

**Published:** 2015-05-01

**Authors:** Saumya Tiwari, Vijaya B. Reddy, Rohit Bhargava, Jaishankar Raman

**Affiliations:** 1 Department of Bioengineering, Beckman Institute for Advanced Science and Technology, University of Illinois at Urbana Champaign, Urbana, Illinois, 61801, United States of America; 2 Department of Pathology, Rush University Medical Center, 1725 West Harrison St, Chicago, Illinois, 60612, United States of America; 3 Department of Bioengineering, Chemistry, Mechanical Science and Engineering, Chemical and Biomolecular Engineering, Electrical and Computer Engineering, Beckman Institute for Advanced Science and Technology and University of Illinois Cancer Center, University of Illinois at Urbana-Champaign, Urbana, Illinois, 61801, United States of America; 4 Cardiac Surgery, Advanced Heart Failure Transplantation & Mechanical Circulatory Support, Rush University Medical Center, 1725 West Harrison St, Chicago, Illinois, 60612, United States of America; Universidad Carlos III of Madrid, SPAIN

## Abstract

Rejection is a common problem after cardiac transplants leading to significant number of adverse events and deaths, particularly in the first year of transplantation. The gold standard to identify rejection is endomyocardial biopsy. This technique is complex, cumbersome and requires a lot of expertise in the correct interpretation of stained biopsy sections. Traditional histopathology cannot be used actively or quickly during cardiac interventions or surgery. Our objective was to develop a stain-less approach using an emerging technology, Fourier transform infrared (FT-IR) spectroscopic imaging to identify different components of cardiac tissue by their chemical and molecular basis aided by computer recognition, rather than by visual examination using optical microscopy. We studied this technique in assessment of cardiac transplant rejection to evaluate efficacy in an example of complex cardiovascular pathology. We recorded data from human cardiac transplant patients’ biopsies, used a Bayesian classification protocol and developed a visualization scheme to observe chemical differences without the need of stains or human supervision. Using receiver operating characteristic curves, we observed probabilities of detection greater than 95% for four out of five histological classes at 10% probability of false alarm at the cellular level while correctly identifying samples with the hallmarks of the immune response in all cases. The efficacy of manual examination can be significantly increased by observing the inherent biochemical changes in tissues, which enables us to achieve greater diagnostic confidence in an automated, label-free manner. We developed a computational pathology system that gives high contrast images and seems superior to traditional staining procedures. This study is a prelude to the development of real time *in situ* imaging systems, which can assist interventionists and surgeons actively during procedures.

## Introduction

The success of cardiac transplantation depends foremost on the immune response to the new implant[1]. The gold standard for identifying allograft rejection is endomyocardial biopsy (EMB)[2]. Endomyocardial biopsy section from a normal heart consists mostly of myocardium which is unoriented and appears red-tan. The tissue section is bordered by the overlying endocardium which is pearly white in appearance[3]. In case of cardiac transplant, an activation of the immune system can cause severe inflammation which can result in transplant rejection and eventual death of patient. Grade of acute cellular rejection, as defined by the revised ISHLT (International Society for Heart & Lung Transplantation) heart biopsy grading scale[4] is determined by the presence of infiltrate and associated myocyte damage. Grade 0 signifies no rejection while grade 2 (mild rejection), 3 (moderate rejection) and 4 (severe rejection) requires assessing the number of foci of infiltrate and associated myocardium damage. Prolonged tissue damage, which could be a result of immune attack, injury or toxins etc. may result in deposition of extracellular matrix components at the site of damage, leading to a condition termed as fibrosis[5–7]. Such an observation of fibrosis is important in assessing myocardium damage in case of allograft rejection. For a detailed description of histopathology associated with cardiac allograph rejection, the readers are directed to available literature[3–5].

In routine cases of monitoring allograft reception, biopsy sections are stained and the inflammatory response is observed, which is predominantly lymphocytic[3]. This approach suffers from inter-observer variability and an inability to quantify accuracy and confidence in data[8,9]. The estimation variance complicates decision-making. For example, misinterpretation of fibrosis through the sub-endocardium can give the erroneous impression of extensive fibrosis[2] and can cause false positives. The subjective nature of histopathological assessment and the apparent potential for errors has long been recognized and debated upon[10,11]. This has led to development of immunohistochemistry for diagnostic purposes by evaluation of specific biomarkers[11,12] but this technique can get affected from variations in sample preparation, fixation procedures, antibody specificity and similar other experimental details[12]. There is a need, therefore, to explore technologies that can make routine histopathological examinations more accurate, consistent, facile and reliable.

As opposed to the standard practice of staining tissue with dyes or molecular imaging of specific epitopes, the emerging technology of chemical imaging can utilize the inherent molecular contrast within samples to provide histologic data. One approach in particular, infrared (IR) spectroscopic imaging, offers strong contrast, high sensitivity and rapid data recording. It has shown potential broadly in biomedical applications for understanding metabolomics and molecular diagnostics[13,14]. Combined with computer algorithms, IR imaging has been used for differentiating between diverse cell types in tissues and for detecting disease[15–17]. Several studies related to cardiovascular systems have reported spectral analysis of tissue and disease in terms of resulting biochemical changes. Infrared imaging has been used to study calcifications in aortic valve[18], for characterizing heart valves [19], studying diabetes induced changes in myocardium and vessels[20–23], for analyzing cardiac extracellular matrix (ECM) remodeling[24]; and ECM and serum components following myocardial infarction[25–28]. While these studies successfully demonstrate differentiation between diseased and healthy tissue via lipid and protein composition and collagen content, a histologic analysis consistent with existing pathology practice is lacking. Characterization of atherosclerotic plaques[29–35] is a step towards clinically-actionable information. However, a practical assay to diagnose conditions and provide actionable information is still lacking. One step in this direction is to utilize digital information obtained from FT-IR spectroscopy and develop a classification protocol which can assign cell identifier value to each pixel on the tissue image. Such classification systems, which require multivariate analysis have been attempted for identification of various cell types in cancer but very little work is found in diseases related to heart[36,37]. Specifically in case of identification of cardiac allograft rejection, we require an automated detection system that has ability to distinguish not only between different cell types but more importantly correctly identify lymphocytes. Identification of lymphocytes is also critically needed as it has potential importance in assessment of many more diseases, for example, identification of tumor infiltrating lymphocytes is also of great interest, and a recent study has sought to identify lymphocytic signature in peripheral blood samples[38]. Another study has utilized unsupervised clustering algorithm to obtain impressive identification of B and T cells in a single patient sample using infrared spectroscopy[39]. However, when analyzing multiple patient samples, accounting for point-to-point variations in the samples and across samples is difficult via unsupervised classifications, leading to reduction in accuracy. The work presented in this manuscript takes this goal of identifying lymphocytes one step further by classifying infiltrating lymphocytes spatially in a biopsy section using supervised classification algorithms of infrared spectroscopy data. Given the complexity and expertise required when conventional pathology is used to diagnose transplant rejection in the heart, we used chemical imaging to see if it could provide the necessary diagnoses and visualizations useful in clinical practice. We utilized differences in the infrared absorbance patterns among different histological classes to develop an automated system where the digital input of IR spectroscopy data yielded a computationally colored image showing different classes similar to what one would obtain using rigorous staining procedures.

## Materials and Methods

### Sample procurement

Written consent was obtained in all patients for study of their archived pathological specimens. The consents were recorded and maintained securely and separately. The consent process for this study was reviewed by the IRB at Rush University Medical Center and approved. All specimens were anonymized, de-identified and no clinical or demographic information was recorded. Thirty five anonymized human EMB sections from ten patients, formalin fixed and paraffin embedded were examined. The biopsies were taken using a bioptome, which is an instrument inserted through the internal jugular vein, and directed under fluoroscopy to be positioned in the right ventricle. The biopsies are then taken as small pieces of tissue, typically measuring 1 mm x 1 mm x1 mm. The section thickness was 5μm. Of the thirty five sections obtained; three sections had to be discarded due to damage to the sections. Out of the 10 patients analyzed, patient 1–5 had no rejection; thus counted as control. Patient 6, 7 and 8 had moderate rejection; and patient 9 and 10 had mild rejection. In current practice, it is very rare to find samples with grade 4 severe rejection, and hence such samples could not be included in the study.

### Sample preparation

Samples were microtomed onto reflective low-emission (Low-E) glass slides for IR imaging. These slides provide a reflective substrate for the sample in IR light but are transparent to visible light. Although when using Low-E slides, the IR beams pass through the sample twice and suffers from distortions in the spectrum[40,41], we have observed that standard preprocessing and treatment of data yields good classification results without performing rigorous corrections for distortions. Albeit using substrates like Calcium Fluoride and Barium Fluoride is preferable, Low-E slides are inexpensive and easy to maintain, making them more practical in clinical environment.

**Fig 1:**
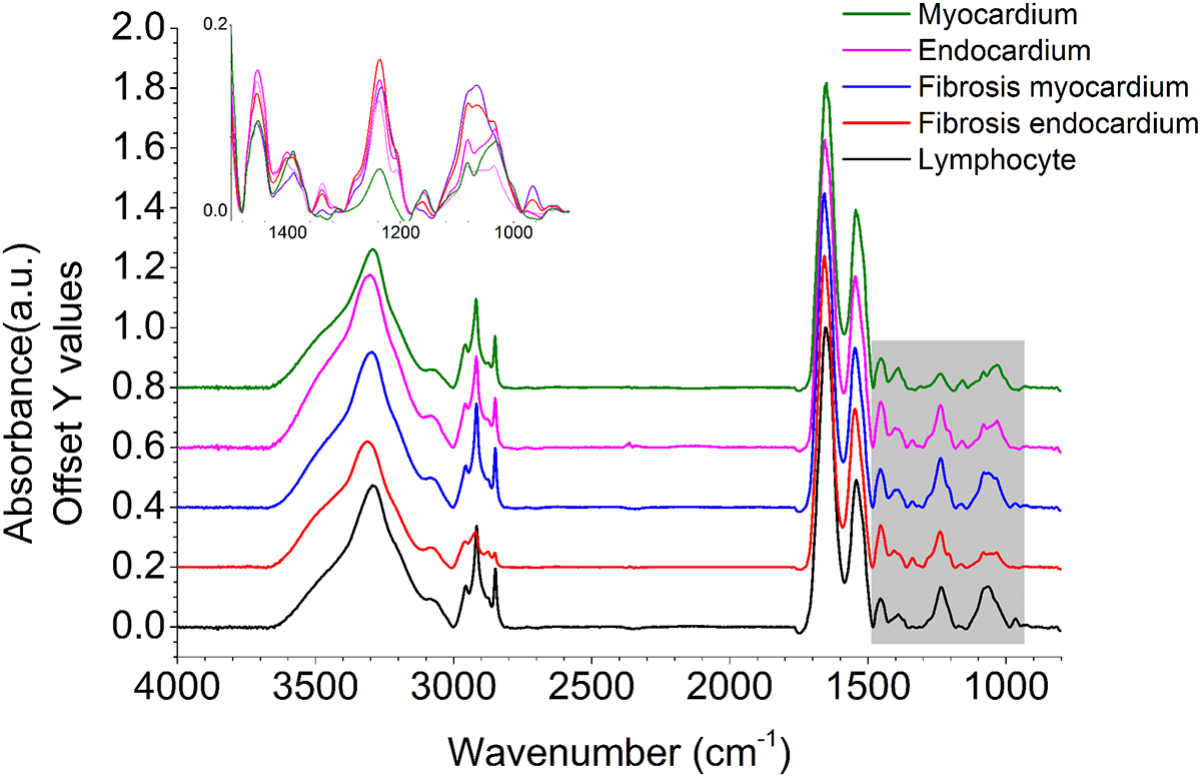
Baseline corrected absorption spectra, normalized using the Amide I peak, for all five classes of cells observed in the study. Important spectral differences observed over the fingerprint spectral region (1500–900 cm^-1^) are highlighted in grey and zoomed in without offset.

Prior to acquiring IR data, paraffin was removed from the samples by washing them twice with hexane and immersion in hexane for 14 hours at room temperature with continuous stirring. Removal of paraffin was evident from the reduction of paraffin-associated CH bending peak at 1464cm^-1^ (Fig 1). Furthermore, the spectral features used in our analysis were extracted from regions which are not affected by paraffin vibrational modes to ensure that any residual paraffin did not interfere with results.

### Fourier transform infrared imaging

De-paraffinized sections were imaged under mid-infrared light on the IR imaging system. FT-IR imaging was performed using a Spotlight 400 system from Perkin Elmer. Spectra were collected using a liquid nitrogen cooled mercury-cadmium-telluride (MCT) 16-element linear array detector. The background was collected on a clear area of low-E slide at 4cm^-1^ resolution using 120 scans for each sample. All images were acquired in reflection mode with 6.25 μm x 6.25 μm pixel size and 4 cm^-1^ spectral resolution with 2cm^-1^ step size using a single interferometer scan with signal to noise ratio (SNR) exceeding 500:1 in all cases. Data was collected over the mid-infrared region and truncated for storage (800cm^-1^ to 4000cm^-1^). The image resolutions were nominally 6.25 μm and 25 μm. Since the samples were large, (smallest dimension being at least 500 μm for every section) and irregularly shaped, each image was acquired by breaking it down to 5–10 smaller rectangular regions and using raster scanning of these parts. Each region was separately focused by the instrument to remove any error due to change in focus and the composite image was stitched back together using ENVI-IDL 4.8(Environment for Visualizing Images-Interactive Data Language). Processing time for a square section of 1mm X 1mm at 6.25 μm, starting with imaging and obtaining computational stain was about 2 hours. Processing time for the same section at 25 μm was about 10 minutes. It has to be kept in mind that this imaging was performed by sweeping through all the wavenumber bands from 4000 cm^-1^ to 800 cm^-1^ at 4 cm^-1^ resolution. After building the classifier, one can realize that only a segment of this range is actually necessary for classification (discussed in results), and thus scanning at discrete frequencies for detection can enable reduction in imaging time by three folds or larger[42,43].

### Hematoxylin and Eosin (H&E) staining

Serial sections were preserved and stained with H&E for initial determination of rejection grade by the pathologist. In addition, after IR imaging was performed on sections, the sections on low-E slides were stained with Hematoxylin and Eosin stains for future comparisons and imaged using Zeiss visible microscope. All the data analysis done in this manuscript used H&E images from same section imaged by infrared spectroscopy and not serial section.

### Data analysis

#### Data pre-processing

Acquired data were imported in ENVI-IDL 4.8 software for analysis. A figure annotating important IR peak assignment is shown in S1 Fig. A very comprehensive table of band assignments of IR spectra of heart tissue is given in this study [20] which can also be referred to. Throughout the analysis, we excluded pixels without protein-characteristic Amide I absorbance since all cells and ECM in this tissue will contain protein (see Fig 1). This was done by setting a threshold of minimum absorbance value corresponding to an absorbance of 0.30, which is at least 10-fold larger than the peak-to-peak noise in the data.

#### Identification of histological features (classes)

With the aim of understanding spectral differences in various components of the section and in order to build a Bayesian classifier for automated classification of the sections, we first built a classification grid comprising of 16 sections by combining data from 16 images in a single file. The breakdown of 16 chosen sections for classification training set was as follows: 3 sections from patient 1, 2 sections each from patient 5, 6,9,10 and 1 section each from patient 2, 3, 4, 7, and 8. Sections were chosen to sufficiently represent each class, namely Myocardium, Endocardium, Fibrosis (Endocardium), Fibrosis (Myocardium) and Lymphocytes and to provide maximum inter-patient diversity to the training set. Remaining sections were used for validation set. The breakdown of validation set was as follows: 2 sections each from patient 3, 4, 5, 6, 7, 8, and 9 and 1 section each from patient 1 and 10.

**Fig 2:**
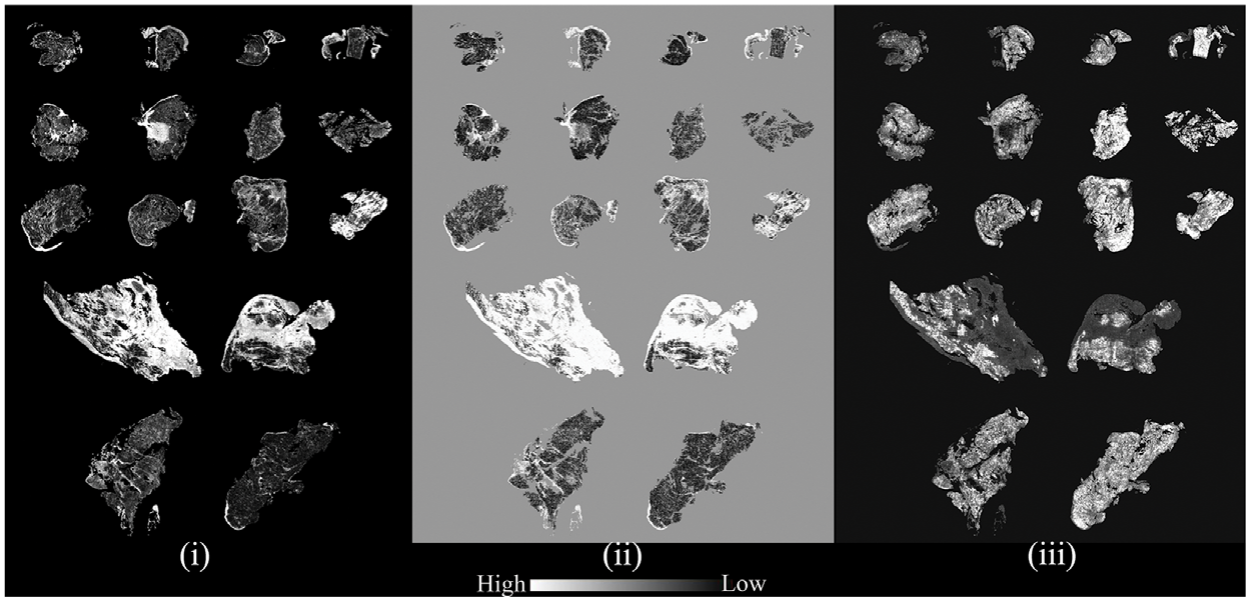
Relative intensities of peak height ratios useful in discriminating classes; examples from metric definitions (i) 1239cm^-1^ to 1652cm^-1^; (ii) 1204cm^-1^ to 1236cm^-1^; (iii) 1027cm^-1^ to 1543cm^-1^.

We then used the peak height of vibrational mode at 1236 cm^-1^ to see contrast between lymphocytes and muscle. The 1236 cm^-1^ peak is associated with CH_2_ wagging vibrations associated with proteins [44,45]; which was found to be useful in prima facie differentiation of different classes (see Fig 2). Five histological classes, namely, Myocardium, Endocardium, Fibrosis (Endocardium), Fibrosis (Myocardium) and Lymphocytes were considered for our analysis. After H&E staining of samples, regions were marked by pathologist as the above classes and this annotation was considered as gold standard[46]. Next, exact same regions were marked in IR images by comparison with pathologist-annotated H&E images from same sections. Care was taken to mark only those regions which clearly belonged to a particular class as seen from H&E images (gold standard, as described earlier [46]). This process yielded approximately 330,000 spectra for training the classification algorithm. For each of the classes, a linear two point correction across peaks of interest or specific peaks was used. The points were fixed for all spectra in the sample. Spectra were normalized to amide I peak (1652 cm^-1^) to account for the variations in sample thickness[47]. We then extracted average spectra for each class, which is shown in Fig 1.

#### Bayesian classification algorithm

Our Bayesian classifier works by determining the likelihood that an unknown pixel belongs to a particular class by using biochemically significant features called metric parameters defined by the user. Each of these parameters can have different weights in the classification process depending on their ability to differentiate between classes. We used a protocol that has previously been established and validated [44,48,49]. Using the spectral differences observed among the classes in training set (shown in Fig 1), we defined a set of 217 parameters using four types of spectral metrics (peak height ratio, peak area to height ratio, peak area to area ratio and center of gravity) to differentiate each class from the others. To begin with, normalized peak heights are considered as parameters for all peaks appearing in absorbance spectrum using peak height ratio with amide I peak. Next, other quantities, peak area to height ratio, peak area to area ratio and center of gravity are defined as metric parameters using peaks in the spectra by manual examination of the differences in spectra between classes. This gave us 217 metric parameters to analyze data with. The significance of these metrics is to reduce data to significant quantities which is readily analyzable[44].

Use of ratios instead of absolute values also ensures that these metric definitions are independent of variability in instrumentation and sample preparation steps. We evaluated these metrics in terms of their ability to separate the classes by using minimum error in identification of class and the area under the curve (AUC) for the Receiver Operating Characteristic (ROC). We further tested the Bayesian classifier built using these parameters on an independent set of sections to evaluate its accuracy in identification of classes. The findings are described in the following section.

## Results and Discussion

### Training

**Fig 3:**
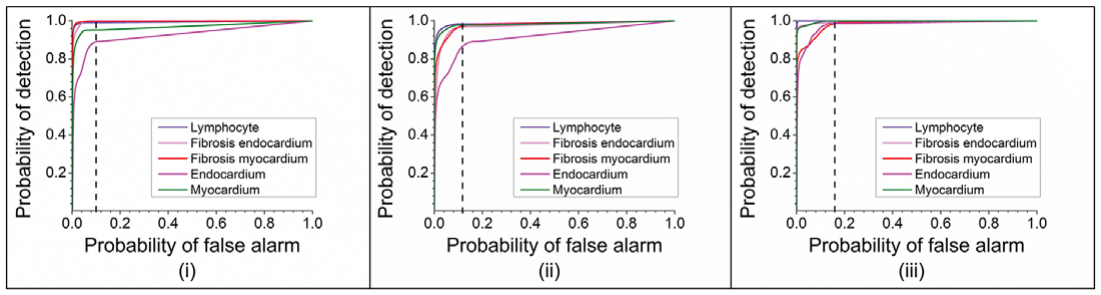
Receiver operating characteristic (ROC) curves demonstrating the accuracy of the classification algorithm (i) Training set at 6.25 μm x 6.25 μm pixel size; (ii) Validation set at 6.25 μm x 6.25 μm pixel size; (iii) Validation set at 25 μm x 25 μm pixel size.

Samples were imaged using IR microscopy and correlated to features in H&E images that were marked by the pathologist’s review as the ground truth. Computerized pattern recognition of IR imaging data from unstained EMB samples led to every tissue pixel being classified into a specific histological class. Compared to the ground truth, the resultant probability of detection at the pixel level for the training set was quite high for lymphocytes (0.991), fibrosis-endocardium (0.999), fibrosis-myocardium (0.997) and myocardium (0.952) and somewhat lower for endocardium (0.860) with approximately 0.10 probability of false alarm (Fig 3(i)). There is a probability of confusing fibrosis with endocardium as evident from confusion matrix shown in Table 1. As can be seen, 48.5% of the tissue identified as endocardium by the pathologist was classified as fibrosis in myocardium and 9.1% of the endocardium was identified as fibrosis in endocardium. It is well known that the nature and structure of endocardium is not well visualized with conventional pathological methods. This may be due to endocardial damage caused by the bioptome and the fact that endocardium is a very thin layer that is easily washed away during preparation. The lower accuracy in this data set also arises from a limited number of endocardium pixels, as partly mitigated by having higher number of pixels in validation set (S1 Table). The issue of endocardium damage during biopsy is unfortunately out of our control. The nature of the biopsy is such that as the bioptome is used along the endocardial surface of the right ventricle, there is inherent damage to the endocardium. This coupled with the fact that the endocardium is a thin, evanescent layer that is easily damaged/disturbed makes endocardial evaluation difficult. Therefore, we have not discussed any precautions for sample preparation. It is important to remember that findings pathognomonic of transplant rejection are not manifested in the endocardium but in the sub-endocardial tissue and in the myocardium. Since our focus here was the identification of transplant rejection, future efforts can be undertaken to refine the data and potentially improve efficiency by better capability instruments for enhanced spatial resolution and faster imaging time. Focused efforts to collect specific tissue components, such as endocardium would give us sample size large enough to accurately characterize these components.

**Table 1:** Confusion matrix for classification for validation data and training data (in parentheses).

	Ground Truth(Percentage)				
Class	Endocardium	Myocardium	Lymphocyte	Fibrosis myocardium	Fibrosis endocardium
Unclassified	3.10	2.25	1.68	1.87	2.21
	(10.90)	(4.80)	(0.90)	(0.30)	(0.10)
Endocardium	**12.74**	0.08	0.00	1.28	0.55
	(12.30)	(0.20)	(0.00)	(0.30)	(0.80)
Myocardium	6.42	**95.91**	0.03	10.11	0.02
	(18.90)	(94.50)	(0.50)	(0.40)	(0.00)
Lymphocyte	0.19	0.02	**82.82**	4.74	0.00
	(0.40)	(0.00)	(85.70)	(13.20)	(0.00)
Fibrosis myocardium	52.91	1.54	15.48	**81.49**	3.53
	(48.50)	(0.50)	(12.90)	(84.80)	(19.10)
Fibrosis endocardium	24.65	0.20	0.00	0.49	**93.69**
	(9.10)	(0.00)	(0.00)	(1.10)	(80.00)
Total	100	100	100	100	100

### Validation

**Fig 4:**
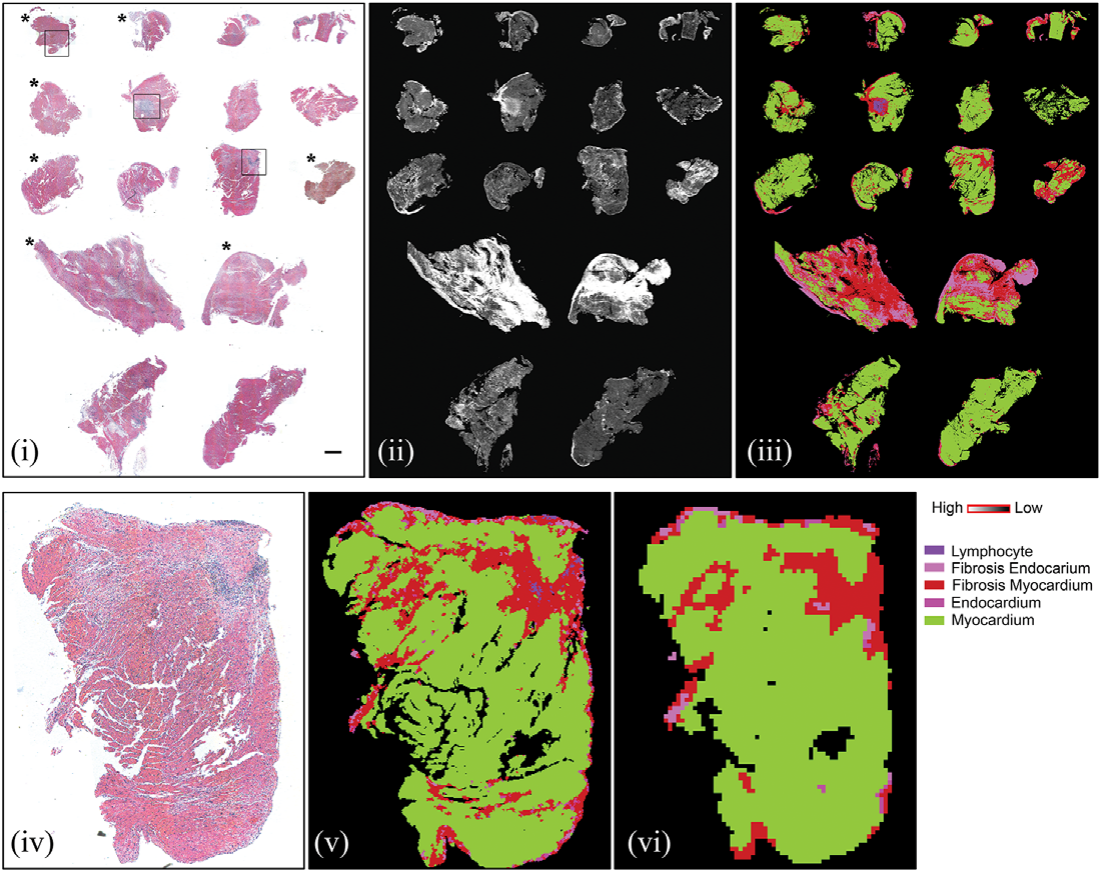
Biopsy section array of 16 samples used for validation. Top panel: (i) H&E stained image of sections (scale bar represents 500μm); Asterisk marked samples showed no rejection in pathologist review. (ii) absorbance at 1236 cm^-1^ demonstrating differences between samples and different cell types; (iii) Classified IR image showing color coded pixels indicating different pathological classes; Bottom panel: Magnified view of one sample from validation set with matched lower spatial resolution IR image. (iv) H&E stained image of section; (v) Classified 6.25 μm x 6.25 μm pixel size IR image; (vi) Classified 25 μm x 25 μm pixel image.

We performed validation using an independent set of 16 samples with approximately 300,000 pixels. Uniformly high probability of detection with low probability of false alarm (0.13) was found (Fig 3(ii)). Comparison of our technique with H&E staining is shown in Fig 4; and comparison from different grades of rejection has been shown for boxed areas from Fig 4 in S2 Fig. Probability of detection at 10% probability of false alarm is provided in Table 2. While achieving significant accuracy, our approach is likely limited by mixed pixels (particularly in regions of lymphocyte infiltration; which is strongly associated with myocyte necrosis and fibrosis) and inclusion of boundary pixels. This limitation is also reflected in identification of endocardium for which we do not have comparatively good representation of pure pixels. Endocardium was not easily visible in the pathology specimens either. While pixel level accuracy may be improved, we achieved accurate identification of the key histopathologic features for decision-making in every sample. Hence, we sought to examine if accurate information could be achieved by speeding up the data acquisition process. Scanning at coarser resolutions can not only make chemical imaging real time but may also lead to higher accuracy due to the higher signal to noise ratio of the detector[50]. Hence, we also collected data at a larger pixel size of 25μm x 25μm (at least 16-fold faster) to evaluate the applicability of the procedure at lower resolution. As seen from Fig 3(iii) and Fig 4, most classes are identified well, but the confidence in data reduces due to larger pixel size. This leads to lower sensitivity which is typical of tradeoff between time required to take image and the resolution achieved[48]. We anticipate that a multi-scale scanning algorithm will be practical when translated to use. The tissue could be scanned in minutes at low resolution and specific areas can be scanned at higher resolution for better accuracy. It is notable that the molecular basis of our histologic approach provides this flexibility, and is truly unique to this technology as we have deployed. In contrast, morphologic analysis of conventional stained tissue is specific to the resolution and is very unlikely to yield similar results. The ability to identify areas of concern with coarse resolution and hone into those areas with high resolution maybe analogous to scanning at low power and then searching the involved areas with high power microscopy. However, the FT-IR imaging based approach is significantly quicker and may be automated quite easily. Presence of lymphocytes in endocardium as well as in nearby myocardium can be checked by looking up neighbors of pixels using simple algorithms; which could also enable us to quantitate foci of infiltrations used for grading rejection. Future efforts can be undertaken to incorporate these ideas and making IR based detection a practical technique by using focal plane array (FPA) detectors for high spatial resolution at faster time frames by utilizing noise reduction[51] techniques.

**Table 2:** Probability of detection at 10% probability of false alarm.

	Training	Validation 6.25 μm	Validation 25μm
Lymphocyte	99.1	98.3	72.9
Fibrosis endocardium	99.9	95.7	93.3
Fibrosis myocardium	99.7	95.9	62
Endocardium	86	85.8	48.4
Myocardium	95.2	97.5	88.7

### Infrared imaging to identify chemical changes in tissue

According to the ISHLT criteria[4], for the sample to be qualified as grade zero (no rejection), there should be no evidence of mononuclear inflammation or myocyte damage. We observed that in all grade 0 cases, there were negligible lymphocyte pixels, and even the lymphocytes that were found were not encroaching in the myocardium. Thus, using IR spectroscopy, it is very straightforward to differentiate between positive (rejected) and negative (not rejected) samples. In addition to histologic identification, biochemical changes undergone by tissues can also be captured using chemical imaging. Since our hypothesis was that the classifier could capture important tissue changes efficiently, we have related specific infrared absorption patterns that we identified from the classifier with previous observations. During fibrosis, ECM components (majorly collagen) are accumulated in the myocardium[52] which is apparent by higher infrared absorption intensity of amide III peak in fibrotic regions[53]. Our classifier correspondingly identified the absorbance at 1236 cm^-1^ (high contribution from collagen[15,45,49,54]) as an important parameter(Tables 3 and 4). Peak due to absorbance at 1236 cm^-1^ / 1239 cm^-1^ is due to the CH_2_ wagging vibrations associated with proteins and is known as amide III peak. However, for the sample being analyzed here, owing to empirical evidences (Fig 2(i)) and observations from past literature discussed above, it can be inferred that major contribution to this peak is coming from collagen. With peaks at 1204 cm^-1^ and 1239 cm^-1^ reflecting the characteristic vibrational modes of collagen proteins-amide III[55] (Fig 2 (i)), a significantly low level was observed in healthy myocardium. Absorbance (1027 cm^-1^ to 1032 cm^-1^) associated with glycogen[56] was decreased at sites of fibrosis (Fig 2(iii)) as previously noted[57]. Hence, this multivariate approach, utilizing multiple biochemical characteristics of tissues, is effective in identifying multiple pathologic conditions.

**Table 3:** List of metric definitions found useful to differentiate classes- peak height ratio; all values are in wavenumber (cm^-1^).

Peak Height Ratio		Peak Height Ratio		Peak Height Ratio	
Peak 1	Peak 2	Peak 1	Peak 2	Peak 1	Peak 2
1389	1236	3315	1236	1204	1236
1027	1065	1163	1236	1239	1652
1239	1543	1163	1065	1236	1543
1389	1452	1452	1543	1236	3300
1239	3300	1389	1652	1389	3300
1389	1065	1452	1236	1027	1543
1405	1236	1155	1452	1032	1236

**Table 4:** List of metric definitions found useful to differentiate classes- peak area to height ratio and center of gravity; all values are in wavenumber (cm^-1^).

Peak area to peak height ratio			Center of gravity			
Left area bound	Right area bound	Peak position	Left bound	Right bound	CG 1	CG 2
1482	1594	1652	1184	1302	1188	1216
1424	1480	1546	1482	1726	1482	1594
1184	1300	1652	984	1144	1016	1048

Together, these results indicate that both the spatial and chemical information can be utilized to identify tissue changes during immune response to the allograft. While we use cardiac allograft rejection as a proof of concept, chemical imaging can be expanded to identify additional cardiac pathologic conditions. Studies show that the false negative rate in identification of myocarditis can be up to 45% due to errors in sampling and sensitivity[58]. Differentiation of lymphocytes from other normal constituents like mast cells, fibroblast nuclei, pericytes and endothelial cells is difficult via visual pathological examination[59]. It has already been shown that IR spectroscopy can identify different cell types[16,60,61]. However, it has been very difficult to classify lymphocytes using earlier supervised classifiers due to low density of lymphocytes in other tissues, and their small size, resulting in problems of mixed pixels at current spatial resolution. It is possible to quantify lymphocyte infiltration in tissue in terms of number of pixels per sample. S1 Table shows the number of pixels marked for each class using gold standard. About 83% of these were correctly identified as lymphocytes by our classifier. This data can be combined with quantification of associated myocyte damage in order to computationally assess the grade of rejection. We are confident that using high definition IR imaging systems[62,63] would enable us to differentiate between even more cell types, making this technique very useful for pathology applications in a variety of conditions.

While manual examination would require presence of lymphocytes in the section (resulting in error due to sampling as well as need to sample multiple times from patient), infrared spectroscopy can potentially detect changes undergone by the tissue which are indicative of transplant rejection even when lymphocytes are not picked in sampling, reducing the error rates, false negatives and avoiding significant trauma to the patient tissue. Apart from chemical information associated with tissues, tissues digitally stained with IR imaging approach are capable of providing a much better contrast and easy quantification of lymphocytes which can greatly reduce the time and effort spent per section by the pathologist.

This report stresses the capabilities of this approach in a complex condition such as cardiac transplant rejection, which traditionally needs careful tissue preparation, multiple stains and review by experienced cardiac pathologists to provide accurate diagnoses. Combined with the speed of the data acquisition and emerging technologies for high speed IR microscopy[42,43], we believe this study opens the path to more rapid tissue assessment much closer to the patient than previously possible. Eventually, intra-operative and *in vivo* imaging can be attempted based on chemical molecular imaging. This can be made possible by touch probe based fiber optic technology on which work is currently under progress. Multiple studies show that spectral in-vivo analysis is promising using probe based instruments and have previously been applied to study atherosclerosis [64–66] and to detect cancer[67,68]. Using attenuated total reflectance (ATR) infrared imaging, mid-infrared light can be used to detect the ailment. Moreover, since this study has already identified specific molecular peaks that can be used for detection, we can now build instruments that operate on discrete frequencies to give even faster detection systems. This is different from near-infrared imaging, which has many pitfalls in making accurate diagnoses[69].

Although the present study has shown excellent promise in terms of on-site detection, we are currently limited by spatial resolution and speed of data acquisition and processing. Spatial resolution used in this study can identify single lymphocytes larger than 10 μm, and lymphocytes smaller than this can be identified in large enough cluster. While the current study can successfully identify grade 0 through grade 4, in order to accurately identify grade 2(mild rejection), one would require much better spatial resolution to identify single lymphocytes. This task can be accomplished in near future using high definition imaging systems which have spatial resolution of the order of single micron, reducing the problem of boundary pixels and enabling us to identify every cell more accurately in tissue. Another challenge faced by pathologists is to identify whether the rejection is cellular or antibody mediated. Good spatial resolution is necessary to identify individual cells and to classify cells that are present in low density in tissues, for example macrophages, basophils; or bacterial cells in case of pathogen infections. As the project expands, we hope to be able to identify many other cell populations in the region such as activated mononuclear cells and pathogens; making spectroscopic analysis of specific cells possible. This could in turn enable us to understand other pathological mechanisms of disease development. While we were limited by speed in terms of imaging and data processing in this study, progress is now being made to reduce data acquisition time by manifolds using discrete infrared spectroscopy [42,43]. The trade-off between the resolution and time can also be improved by the use of FPA detectors, using which large areas can be measured at higher resolutions at faster time frames, and which are becoming more and more amenable. These advances further go on to show that IR imaging provides a potential approach for next generation histology procedures that are highly precise and accurate while the automation can lead to better decision making closer to the patient. This could be done within a very short period of time; thereby reducing the work load on pathologist and bringing smart detection devices to surgery suites.

## Conclusion

The chemical molecular imaging approach offers numerous advantages over traditional sample examination techniques, providing a new avenue for clinical diagnosis. Chemical information, along with morphologic and architectural tissue information provides for a comprehensive analysis of tissue. Computer algorithms allow us to dispense with staining and pathological recognition is aided by color coded images. In this study, we have shown an example of how chemical imaging can be applied in cardiac tissues to achieve automated pathology while providing a high probability of detection and low probability of false alarm. We identified specific spectral characteristics which related to the biochemical changes undergone by the tissue which could be used for chemical detection of rejection. In future, we can make this even more extensive by differentiating between acute cellular rejection and Quilty lesions. This is the first study to show that the chemical molecular imaging approach can be used to diagnose complex cardiac conditions, with results equivalent to and probably superior to conventional pathology.

This technique would also be useful in identifying other cell populations that can be present in cardiovascular environment such as activated immune cells, antibody mediated rejection and bacterial infections to name a few. It is also possible to integrate this digital data with patient history to provide an even more nuanced scientific assessment of disease and prognosis. The idea here is to kick start the development of an approach which can give an all-encompassing rapid diagnosis at the site of collection of sample without stains and more importantly, assist during surgery for identification of diseased and problem areas in the heart & vasculature.

## Supporting Information

S1 Fig IR peak assignments for tissue. (TIF)

S2 Fig Computationally stained infrared image compared with H&E image at various grades of rejection. Arrows show lymphocytic infiltration. Top panel: No rejection; Middle panel: Mild rejection; Bottom panel: Moderate rejection.
(TIF)

S1 Table Number of pixels for each class in training and validation sets. (XLS)

## References

[1] Patel JK, Kittleson M, Kobashigawa JA. Cardiac allograft rejection. Surgeon. Elsevier Ltd; 2011;9: 160–7. 10.1016/j.surge.2010.11.023 21550522

[2] Fenoglio JJ, Marboe CC. Endomyocardial biopsy: an overview. Hum Pathol. WB Saunders; 1987;18: 609–612. 10.1016/s0046-8177(87)80361-1 3297991

[3] Stone JR, Basso C, Baandrup UT, Bruneval P, Butany J, Gallagher PJ, et al Recommendations for processing cardiovascular surgical pathology specimens: a consensus statement from the Standards and Definitions Committee of the Society for Cardiovascular Pathology and the Association for European Cardiovascular Pathology. Cardiovasc Pathol. Elsevier Inc.; 2012;21: 2–16. 10.1016/j.carpath.2011.01.001 21353600

[4] Stewart S, Winters G, Fishbein M, Tazelaar H, Kobashigawa J, Abrams J, et al Revision of the 1990 working formulation for the standardization of nomenclature in the diagnosis of heart rejection. J Hear Lung Transplant. 2005;24: 1710–20. 10.1016/j.healun.2005.03.019 16297770

[5] Lange LG, Schreiner GF. Mechanisms of disease. N Engl J Med. 1994;330: 1129–1135. 8133856 10.1056/NEJM199404213301607

[6] Friedman SL. Mechanisms of disease: Mechanisms of hepatic fibrosis and therapeutic implications. Nat Clin Pract Gastroenterol Hepatol. 2004;1: 98–105. 10.1038/ncpgasthep0055 16265071

[7] Kumar V, Abbas AK, Fausto N, Aster J. Tissue Renewal, Regeneration, and Repair Pathologic Basis of Disease. 8th ed Philadelphia: Elsevier; 2010 pp. 79–110.

[8] Shanes JG, Ghali J, Billingham ME, Ferrans VJ, Fenoglio JJ, Edwards WD, et al Interobserver variability in the pathologic interpretation of endomyocardial biopsy results. Circulation. 1987;75: 401–405. 10.1161/01.CIR.75.2.401 3802444

[9] Hahn EA, Hartz VL, Moon TE, O’Connell JB, Herskowitz A, McManus BM, et al The Myocarditis Treatment Trial: design, methods and patients enrollment. Eur Heart J. 1995;16 Suppl O: 162–167. 8682088 10.1093/eurheartj/16.suppl_o.162

[10] Edwards WD. Current problems in establishing quantitative histopathologic criteria for the diagnosis of lymphocytic myocarditis by endomyocardial biopsy. Heart Vessels Suppl. 1985;1: 138–142. 10.1007/BF02072381 3843576

[11] Hammond MEH, Stehlik J, Snow G, Renlund DG, Seaman J, Dabbas B, et al Utility of histologic parameters in screening for antibody-mediated rejection of the cardiac allograft: A study of 3,170 biopsies. J Hear Lung Transplant. 2005;24: 2015–2021. 10.1016/j.healun.2005.08.014 16364843

[12] Walker RA. Quantification of immunohistochemistry—Issues concerning methods, utility and semiquantitative assessment I. Histopathology. 2006;49: 406–410. 10.1111/j.1365-2559.2006.02514.x 16978204

[13] Ellis DI, Goodacre R. Metabolic fingerprinting in disease diagnosis: biomedical applications of infrared and Raman spectroscopy. Analyst. 2006;131: 875–85. 10.1039/b602376m 17028718

[14] Petrich W. Mid-infrared and raman spectroscopy for medical diagnostics. Appl Spectrosc Rev. 2001;36: 181–237. 10.1081/A

[15] Holton SE, Walsh MJ, Kajdacsy-Balla A, Bhargava R. Label-free characterization of cancer-activated fibroblasts using infrared spectroscopic imaging. Biophys J. Biophysical Society; 2011;101: 1513–21. 10.1016/j.bpj.2011.07.055 21943433 PMC3177049

[16] Walsh MJ, Reddy RK, Bhargava R, Member A. Label-Free Biomedical Imaging With Mid-IR Spectroscopy. IEEE J Sel Top quantum Electron. 2012;18: 1502–1513.

[17] Nallala J, Piot O, Diebold M-D, Gobinet C, Bouché O, Manfait M, et al Infrared imaging as a cancer diagnostic tool: introducing a new concept of spectral barcodes for identifying molecular changes in colon tumors. Cytometry A. 2013;83: 294–300. 10.1002/cyto.a.22249 23303722

[18] Dritsa V, Pissaridi K, Koutoulakis E, Mamarelis I, Kotoulas C, Anastassopoulou J. An infrared spectroscopic study of aortic valve. A possible mechanism of calcification and the role of magnesium salts. In Vivo. 2014;28: 91–8. 24425841

[19] Jastrzebska M, Zalewska-Rejdak J, Mróz I, Barwinski B, Wrzalik R, Kocot A, et al Atomic force microscopy and FT-IR spectroscopy investigations of human heart valves. Gen Physiol Biophys. 2006;25: 231–244. 17197723

[20] Toyran N, Lasch P, Naumann D, Turan B, Severcan F. Early alterations in myocardia and vessels of the diabetic rat heart: an FTIR microspectroscopic study. Biochem J. 2006;397: 427–436. 10.1042/BJ20060171 16719841 PMC1533317

[21] Toyran N, Severcan F, Severcan M, Turan B. Investigation of diabetes-induced effect on apex of rat heart myocardium by using cluster analysis and neural network approach: An FTIR study. Spectroscopy. 2007;21: 269–278. 10.1155/2007/269618

[22] Majzner K, Wrobel TP, Fedorowicz A, Chlopicki S, Baranska M. Secondary structure of proteins analyzed ex vivo in vascular wall in diabetic animals using FT-IR spectroscopy. Analyst. 2013;138: 7400–10. 10.1039/c3an00455d 24179990

[23] Birarda G, Holman EA, Fu S, Weikel K, Hu P. Synchrotron infrared imaging of advanced glycation endproducts (AGEs) in cardiac tissue from mice fed high glycemic diets. Biomed Spectrosc Imaging. 2013;2: 301–315. 10.3233/BSI-130057 26500847 PMC4617198

[24] Gough KM, Zelinski D, Wiens R, Rak M, Dixon IMC. Fourier transform infrared evaluation of microscopic scarring in the cardiomyopathic heart: Effect of chronic AT1 suppression. Anal Biochem. 2003;316: 232–242. 10.1016/S0003-2697(03)00039-3 12711345

[25] Cheheltani R, Wang B, Sabri A, Pleshko N, Kiani M. Fourier Transform Infrared Imaging Spectroscopy of Collagen Deposition after Myocardial Infarction Bioengineering Conference (NEBEC), 2012 38th Annual Northeast. IEEE; 2012 pp. 305–306.

[26] Petrich W, Lewandrowski KB, Muhlestein JB, Hammond MEH, Januzzi JL, Lewandrowski EL, et al Potential of mid-infrared spectroscopy to aid the triage of patients with acute chest pain. Analyst. 2009;134: 1092–1098. 10.1039/b820923e 19475134

[27] Wetzel DL, Post GR, Lodder RA. Synchrotron infrared microspectroscopic analysis of collagens I, III, and elastin on the shoulders of human thin-cap fibroatheromas. Vib Spectrosc. 2005;38: 53–59. 10.1016/j.vibspec.2005.02.029

[28] Lui K, Jackson M, Sowa MG, Ju H, Dixon IM, Mantsch HH. Modification of the extracellular matrix following myocardial infarction monitored by FTIR spectroscopy. Biochim Biophys Acta. 1996;1315: 73–7. 8608173 10.1016/0925-4439(95)00118-2

[29] Manoharan R, Baraga JJ, Rava RP, Dasari RR, Fitzmaurice M, Feld MS. Biochemical analysis and mapping of atherosclerotic human artery using FT-IR microspectroscopy. Atherosclerosis. 1993;103: 181–193. 10.1016/0021-9150(93)90261-R 8292094

[30] Baraga JJ, Feld MS, Rava RP. Detection of Atherosclerosis in Human Artery by Mid-Infrared Attenuated Total Reflectance. Appl Spectrosc. 1991;45: 709–711. 10.1366/0003702914337047

[31] Wrobel TP, Mateuszuk L, Chlopicki S, Malek K, Baranska M. Imaging of lipids in atherosclerotic lesion in aorta from ApoE/LDLR-/- mice by FT-IR spectroscopy and Hierarchical Cluster Analysis. Analyst. 2011;136: 5247 10.1039/c1an15311k 22007352

[32] Kodali DR, Small DM, Powell J, Krishnan K. Infrared Micro-imaging of Atherosclerotic Arteries. Appl Spectrosc. 1991;45: 1310–1317. 10.1366/0003702914335878

[33] Wrobel TP, Majzner K, Baranska M. Protein profile in vascular wall of atherosclerotic mice analyzed ex vivo using FT-IR spectroscopy. Spectrochim Acta—Part A Mol Biomol Spectrosc. Elsevier B.V.; 2012;96: 940–945. 10.1016/j.saa.2012.07.103 22944148

[34] Moreno PR. Detection of Lipid Pool, Thin Fibrous Cap, and Inflammatory Cells in Human Aortic Atherosclerotic Plaques by Near-Infrared Spectroscopy. Circulation. 2002;105: 923–927. 10.1161/hc0802.104291 11864919

[35] Römer TJ, Brennan JF, Fitzmaurice M, Feldstein ML, Deinum G, Myles JL, et al Histopathology of human coronary atherosclerosis by quantifying its chemical composition with Raman spectroscopy. Circulation. Am Heart Assoc; 1998;97: 878–885. 10.1161/01.cir.97.9.878 9521336

[36] Wang L, Chapman J, Palmer RA, van Ramm O, Mizaikoff B. Classification of atherosclerotic rabbit aorta samples by mid-infrared spectroscopy using multivariate data analysis. J Biomed Opt. 2007;12: 024006 10.1117/1.2714030 17477721

[37] Toyran N, Severcan F, Severcan M, Turan B. Effects of selenium supplementation on rat heart apex and right ventricle myocardia by using FTIR spectroscopy: A cluster analysis and neural network approach. Food Chem. 2008;110: 590–597. 10.1016/j.foodchem.2008.02.044

[38] Wald N, Legat A, Meyer C, Speiser DE, Goormaghtigh E. An infrared spectral signature of human lymphocyte subpopulations from peripheral blood. Analyst. Royal Society of Chemistry; 2015; 10.1039/c4an02247e 25553786

[39] Krafft C, Salzer R, Soff G, Meyer-Hermann M. Identification of B and T cells in human spleen sections by infrared microspectroscopic imaging. Cytom Part A. 2005;64: 53–61. 10.1002/cyto.a.20117 15729712

[40] Bassan P, Lee J, Sachdeva A, Pissardini J, Dorling KM, Fletcher JS, et al The inherent problem of transflection-mode infrared spectroscopic microscopy and the ramifications for biomedical single point and imaging applications. Analyst. 2013; 144–157. 10.1039/c2an36090j 23099638

[41] Filik J, Frogley MD, Pijanka JK, Wehbe K, Cinque G. Electric field standing wave artefacts in FTIR micro-spectroscopy of biological materials. Analyst. 2012;137: 853 10.1039/c2an15995c 22231204

[42] Yeh K, Kenkel S, Liu J, Bhargava R. Fast Infrared Chemical Imaging with a Quantum Cascade Laser. Anal Chem. 2015; 10.1021/ac5027513 PMC4287837 25474546

[43] Kole MR, Reddy RK, Schulmerich MV, Gelber MK, Bhargava R. Discrete frequency infrared microspectroscopy and imaging with a tunable quantum cascade laser. Anal Chem. 2012;84: 10366–72. 10.1021/ac302513f 23113653 PMC3514576

[44] Bhargava R, Fernandez DC, Hewitt SM, Levin IW. High throughput assessment of cells and tissues: Bayesian classification of spectral metrics from infrared vibrational spectroscopic imaging data. Biochim Biophys Acta. 2006;1758: 830–45. 10.1016/j.bbamem.2006.05.007 16822477

[45] Jackson M, Choo LP, Watson PH, Halliday WC, Mantsch HH. Beware of connective tissue proteins: assignment and implications of collagen absorptions in infrared spectra of human tissues. Biochim Biophys Acta. 1995;1270: 1–6. 7827129 10.1016/0925-4439(94)00056-v

[46] Fernandez DC, Bhargava R, Hewitt SM, Levin IW. Infrared spectroscopic imaging for histopathologic recognition. Nat Biotechnol. 2005;23: 469–74. 10.1038/nbt1080 15793574

[47] Martin FL, Kelly JG, Llabjani V, Martin-Hirsch PL, Patel II, Trevisan J, et al Distinguishing cell types or populations based on the computational analysis of their infrared spectra. Nat Protoc. 2010;5: 1748–1760. 10.1038/nprot.2010.133 21030951

[48] Bhargava R. Towards a practical Fourier transform infrared chemical imaging protocol for cancer histopathology. Anal Bioanal Chem. 2007;389: 1155–69. 10.1007/s00216-007-1511-9 17786414

[49] Pounder FN, Reddy RJ, Walsh MJ, Bhargava R. Validating the cancer diagnosis potential of mid-infrared spectroscopic imaging Coté GL , editor Proc SPIE. 2009;7186: 71860F–71860F–9. 10.1117/12.810122

[50] Bhargava R, Levin IW. Fourier transform infrared imaging: theory and practice. Anal Chem. 2001;73: 5157–67. 11721913 10.1021/ac010380m

[51] Reddy RK, Bhargava R. Accurate histopathology from low signal-to-noise ratio spectroscopic imaging data. Analyst. 2010;135: 2818–25. 10.1039/c0an00350f 20830324

[52] Wei L. Immunological aspect of cardiac remodeling: T lymphocyte subsets in inflammation-mediated cardiac fibrosis. Exp Mol Pathol. 2011;90: 74–8. 10.1016/j.yexmp.2010.10.004 20965166

[53] Wang Q, Sanad W, Miller LM, Voigt A, Klingel K, Kandolf R, et al Infrared imaging of compositional changes in inflammatory cardiomyopathy. Vib Spectrosc. 2005;38: 217–222. 10.1016/j.vibspec.2005.02.011

[54] Fung MFK, Senterman MK, Mikhael NZ, Lacelle S, Wong PTT. Pressure-tuning Fourier Transform Infrared Spectroscopic Study of Carcinogenesis in Human Endometrium. Biospectroscopy. 1996;2: 155–165.

[55] Sionkowska A, Wisniewski M, Skopinska J, Kennedy CJ, Wess TJ. Molecular interactions in collagen and chitosan blends. Biomaterials. 2004;25: 795–801. 10.1016/S0142-9612(03)00595-7 14609668

[56] Parker FS. Applications of infrared spectroscopy in biochemistry, biology and medicine 1971.

[57] Burch GE, Sun S-C, Sohal RS, Chu K-C, Colcolough HL. Diphtheritic Myocarditis. Am J Cardiol. 1968;21: 261–268. 4865613 10.1016/0002-9149(68)90328-7

[58] Hauck AJ, Kearney DL, Edwards WD. Evaluation of postmortem endomyocardial biopsy specimens from 38 patients with lymphocytic myocarditis: implications for role of sampling error. Mayo Clinic Proceedings. 1989 pp. 1235–1245. 2593714 10.1016/s0025-6196(12)61286-5

[59] Thomas Aretz H. Myocarditis: The Dallas criteria. Hum Pathol. W. B. Saunders Co.; 1987;18: 619–624. 10.1016/S0046-8177(87)80363-5 3297992

[60] Wood BR, Quinn MA, Tait B, Ashdown M, Hislop T, Romeo M, et al FTIR microspectroscopic study of cell types and potential confounding variables in screening for cervical malignancies. Biospectroscopy. 1998;4: 75–91. 10.1002/(SICI)1520-6343(1998)4:2<75::AID-BSPY1>3.0.CO;2-R 9557903

[61] German MJ, Hammiche A, Ragavan N, Tobin MJ, Cooper LJ, Matanhelia SS, et al Infrared Spectroscopy with Multivariate Analysis Potentially Facilitates the Segregation of Different Types of Prostate Cell. Biophys J. Elsevier; 2006;90: 3783–3795. 10.1529/biophysj.105.077255 16500983 PMC1440759

[62] Nasse MJ, Walsh MJ, Mattson EC, Reininger R, Kajdacsy-Balla A, Macias V, et al High-resolution Fourier-transform infrared chemical imaging with multiple synchrotron beams. Nat Methods. 2011;8: 413–6. 10.1038/nmeth.1585 21423192 PMC3877692

[63] Walsh MJ, Mayerich D, Kajdacsy-Balla A, Bhargava R. High-resolution mid-infrared imaging for disease diagnosis. Mahadevan-Jansen A, Petrich W, editors. 2012;8219: 82190R 10.1117/12.909339

[64] Motz JT, Gandhi SJ, Scepanovic OR, Haka AS, Kramer JR, Dasari RR, et al Real-time Raman system for in vivo disease diagnosis. J Biomed Opt. 2005;10: 031113 10.1117/1.1920247 16229638

[65] Motz JT, Fitzmaurice M, Miller A, Gandhi SJ, Haka AS, Galindo LH, et al In vivo Raman spectral pathology of human atherosclerosis and vulnerable plaque. J Biomed Opt. 2006;11: 021003 10.1117/1.2190967 16674178

[66] Buschman HP, Marple ET, Wach ML, Bennett B, Schut TC, Bruining HA, et al In vivo determination of the molecular composition of artery wall by intravascular Raman spectroscopy. Anal Chem. 2000;72: 3771–3775. 10959962 10.1021/ac000298b

[67] Mackanos MA, Contag CH. Fiber-optic probes enable cancer detection with FTIR spectroscopy. Trends Biotechnol. Elsevier Ltd; 2010;28: 317–323. 10.1016/j.tibtech.2010.04.001 20452071

[68] Mackanos MA, Hargrove J, Wolters R, Du CB, Friedland S, Soetikno RM, et al Use of an endoscope-compatible probe to detect colonic dysplasia with Fourier transform infrared spectroscopy. J Biomed Opt. 2014;14: 044006 10.1117/1.3174387 PMC3232016 19725718

[69] Ferrari M, Mottola L, Quaresima V. Principles, techniques, and limitations of near infrared spectroscopy. Can J Appl Physiol. 2004;29: 463–487. 15328595 10.1139/h04-031

