# Supplementary figures and images for: Computational Chemical Imaging for Cardiovascular Pathology: Chemical Microscopic Imaging Accurately Determines Cardiac Transplant Rejection
Source: PLoS One. 2015 May 1;10(5):e0125183. doi: 10.1371/journal.pone.0125183 (PMC4416885; doi:10.1371/journal.pone.0125183)

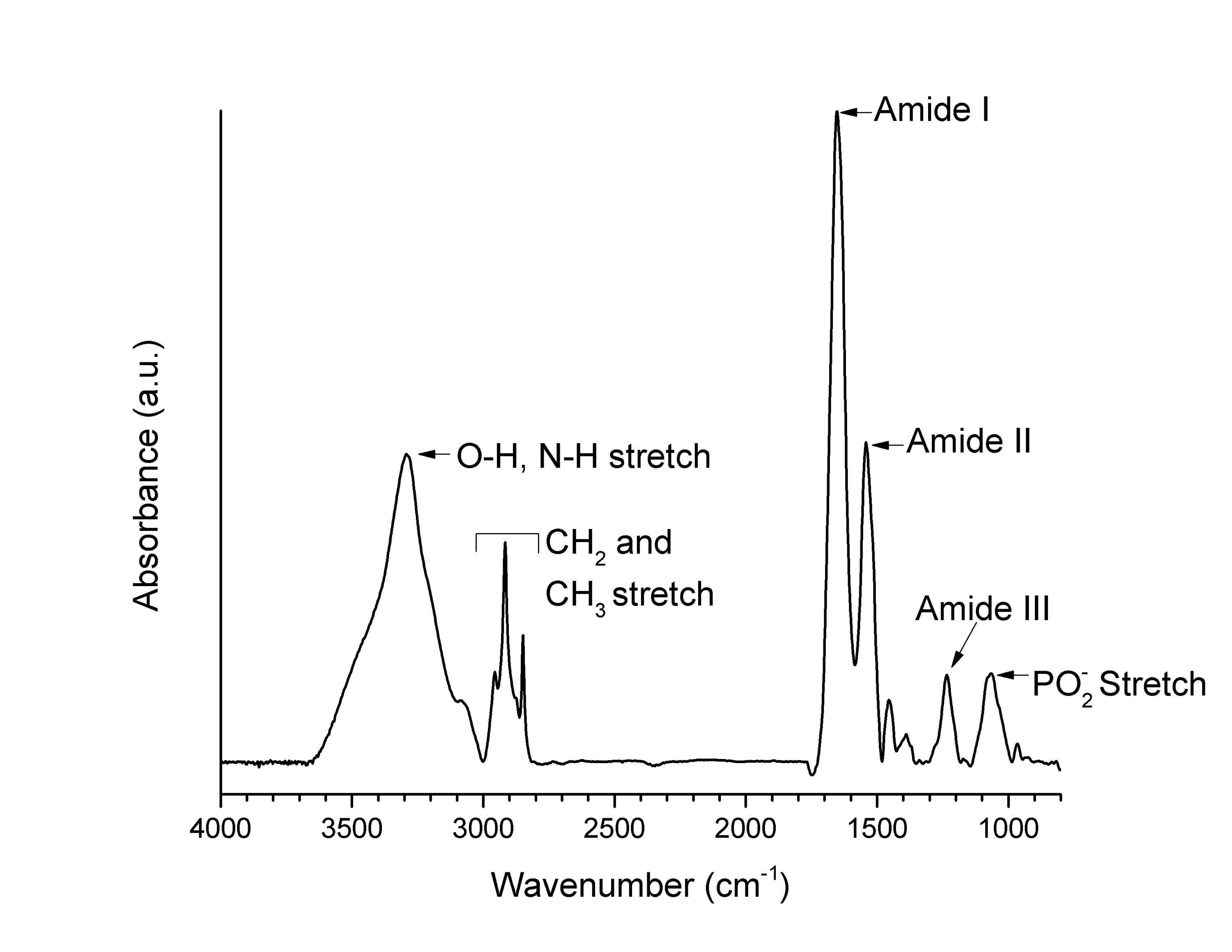

Supplement: S1 Fig — (TIF) [file pone.0125183.s001.tif]

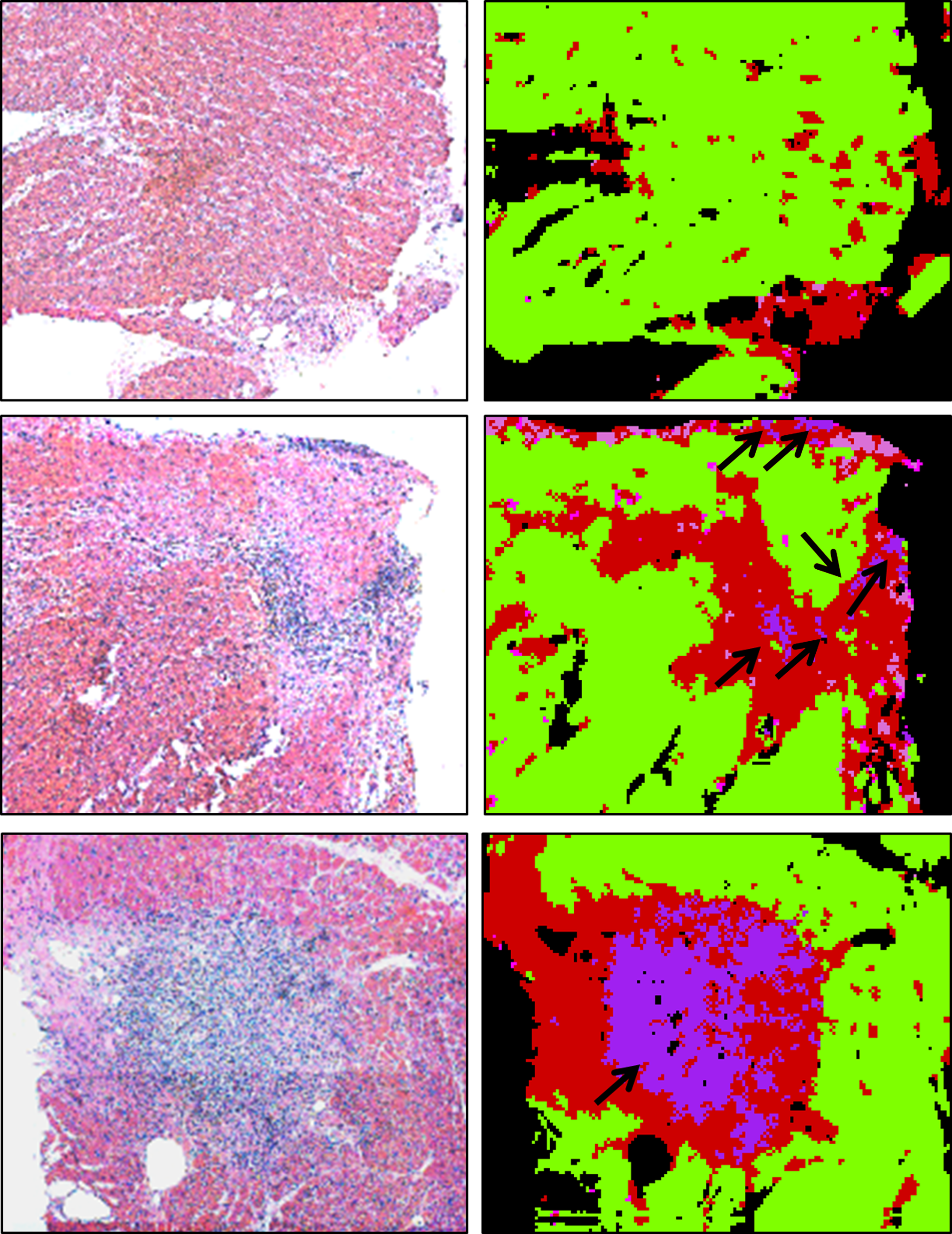

Supplement: S2 Fig — Arrows show lymphocytic infiltration. Top panel: No rejection; Middle panel: Mild rejection; Bottom panel: Moderate rejection. (TIF) [file pone.0125183.s002.tif]
